# *StSUT2* Regulates Cell Wall Architecture and Biotic Stress Responses in Potatoes (*Solanum tuberosum*)

**DOI:** 10.3390/plants14182941

**Published:** 2025-09-22

**Authors:** Huiling Gong, Hongmei Li, Chenxia Wang, Qian Kui, Leonce Dusengemungu, Xia Cai, Zaiping Feng

**Affiliations:** 1 School of Life Sciences and Technology, Lanzhou University of Technology, Lanzhou 730050, China; lhmpdx@163.com (H.L.); 13893406602@163.com (C.W.); 19909426646@163.com (Q.K.); caixia@lut.edu.cn (X.C.); fengzaiping@163.com (Z.F.); 2 African Agricultural Technology Foundation, RAB Rubirizi Station, Kigali P.O. Box 5016, Rwanda; l.dusengemungu@aatf-africa.org

**Keywords:** RNA interference, sucrose transporter, *Fusarium sulphureum*, cell wall composition, *Ralstonia solanacearum*

## Abstract

Plant sucrose transporters (SUTs) are essential membrane proteins that mediate sucrose phloem loading in source tissues and unloading in sink tissues. In addition to their role in carbohydrate partitioning, SUTs have been implicated in plant responses to both biotic and abiotic stresses. Our previous research demonstrated that silencing *StSUT2* in potatoes (*Solanum tuberosum*) affects plant growth, flowering time, and tuber yield, with transcriptomic analysis suggesting its involvement in cell wall metabolic pathways. In this study, we further investigated the effects of *StSUT2* inhibition on the cell wall structure and biotic stress response of potatoes. Transmission electron microscopy revealed that the tuber cell wall thickness of the *StSUT2* RNA interference (RNAi) line *RNAi-2* was reduced by 7.8%, and the intercellular space was increased by 214% compared with the wild-type plants. Biochemical analyses showed that *StSUT2* silencing significantly decreased cellulose, hemicellulose, and lignin contents in both the leaves and tubers, e.g., tuber cellulose reduced by up to 20.1%, while pectin levels remained unaffected, with distinct effects on source leaves and sink tubers’ organs. Additionally, activities of cellulase, xyloglucan glycosyltransferase/hydrolase XTH, and polygalacturonase were elevated in RNAi lines, e.g., leaf cellulase increased by 43.3%, whereas the pectinase activity was unchanged. Pathogen inoculation assays demonstrated that *StSUT2* RNAi lines were more susceptible to *Ralstonia solanacearum* bacterial wilt and *Fusarium sulphureum* dry rot, showing larger leaf lesions, wider tuber necrotic plaques, and severe seedling wilting. These findings demonstrate that silencing *StSUT2* regulates the cell wall structure, composition, and the activity of cell wall-degrading enzymes, thereby reducing the plant’s resistance to fungal and bacterial pathogens.

## 1. Introduction

Sucrose is the main form of carbohydrate transported from source to sink tissues in higher plants. The efficient allocation of sucrose is crucial for plant growth, development, and responses to environmental stimuli. Two primary pathways mediate sucrose translocation: the symplastic pathway, which utilizes plasmodesmata for intercellular movement, and the apoplastic pathway, which involves energy-dependent membrane transporters such as sucrose transporters (SUTs). SUTs, also referred to as sucrose/H^+^ symporters (SUCs), are membrane-bound proteins responsible for sucrose loading into the phloem in source tissues and unloading in sink tissues [1]. Based on sequence homology and functional properties, SUTs are classified into five subfamilies, each playing distinct roles in carbohydrate partitioning [2].

SUTs do more than mediate sugar allocation, they also play roles in regulating flowering, seed development, stress responses, and interactions with microbes. For example, suppression of the *SlSUT2* in the tomato resulted in increased mycorrhizal colonization, indicating that SUT2 may limit symbiotic interactions by retrieving sucrose from the apoplast [3]. Moreover, *SlSUT2* was shown to interact with brassinosteroid signaling components such as *BAK1* and *MSBP1*, linking sugar transport to hormone signaling [4]. In transgenic tomato lines with reduced *SlSUT2* expression, susceptibility to the pathogen “Candidatus Phytoplasma solani” increased significantly, as indicated by the higher bacterial DNA and rRNA accumulation in leaves [5]. In rice, the CRISPR/Cas9-mediated knockout of *OsSUT2* led to the decreased resistance to fungal pathogens like Saccharomyces cerevisiae, suggesting a conserved role of SUT2 in defense [6].

Recent studies further demonstrate the critical role of SUTs in crop defense, particularly through their influence on cell wall dynamics and pathogen resistance. In maize, ZmSUT2 has been shown to regulate sucrose allocation to developing kernels, with its suppression leading to reduced lignin and cellulose content in cell walls, increasing the susceptibility to *Fusarium verticillioides* [7]. Similarly, in wheat, TaSUT2 modulates the sucrose transport to grains and contributes to cell wall reinforcement, with mutants exhibiting an enhanced susceptibility to *Fusarium graminearum* due to altered hemicellulose levels [8]. These findings, alongside studies in tomato and rice, highlight a conserved role for SUT2 in linking sucrose transport to defense mechanisms across diverse crops. By regulating carbon availability for cell wall biosynthesis, SUTs influence the physical and biochemical barriers that limit pathogen ingress, suggesting their potential as targets for enhancing crop resilience [9]. These studies provide a broader context for understanding the multifaceted roles of SUTs in plant immunity and stress responses.

The potato (*Solanum tuberosum* L.) is the fourth most important food crop globally and a vital source of nutrition. In China and other regions, potato yields are substantially affected by biotic stresses, including *Ralstonia solanacearum* (causing bacterial wilt), *Fusarium sulphureum* (causing dry rot), and Phytophthora infestans (causing late blight). These pathogens compromise plant health and reduce tuber quality and marketability [10,11]. Plant resistance to pathogens is closely associated with the composition and structure of the cell wall, which acts as a primary defense barrier. Changes in lignin, cellulose, hemicellulose, and pectin levels can influence the plant’s ability to withstand microbial attacks [12,13].

Three *SUT* genes have been identified in the potato, namely *StSUT1*, *StSUT2*, and *StSUT4* [14]. At the molecular level, potato sucrose transporters StSUT1, StSUT2, and StSUT4 exhibit distinct sequence and structural characteristics [15]. StSUT1, a high-affinity transporter, shows strong expression in source leaves, with 12 transmembrane domains optimized for efficient phloem loading [16]. StSUT2, a low-affinity transporter, has a unique N-terminal extension and divergent cytoplasmic loops, sharing only 50–60% amino acid identity with StSUT1 and StSUT4, and is expressed in both source and sink tissues, suggesting roles in signaling and sucrose partitioning [17]. StSUT4, with a moderate affinity, contains specific C-terminal motifs and is predominantly expressed in sink tissues, influencing circadian rhythms, ethylene production, and tuberization through the modulation of sucrose allocation [18,19]. These molecular distinctions, particularly StSUT2′s unique structural motifs, likely enable its interaction with stress-related pathways, setting it apart from the transport-focused StSUT1 and development-oriented StSUT4.

*StSUT2*, originally proposed to function as a sucrose sensor [17], has more recently been described as a weak or low-affinity sucrose transporter with broader regulatory roles [15]. While its capacity for direct sucrose transport remains limited compared to canonical SUT1-type proteins, accumulating evidence supports its function as a regulator of sucrose status and signaling, potentially integrating metabolic and defense pathways [20]. However, its role in modulating plant immunity and cell wall dynamics remains underexplored.

Our recent study revealed that RNA interference (RNAi)-mediated silencing of *StSUT2* caused profound phenotypic changes, including reduced plant height, altered flowering time, and diminished tuber yield [15]. Transcriptomic profiling of *StSUT2* RNAi line showed significant enrichment of differentially expressed genes associated with cell wall metabolism, suggesting a novel regulatory role of *StSUT2* in cell wall remodeling [15]. These findings raise important questions: does *StSUT2* influence the structural integrity of the cell wall? Does it contribute to resistance mechanisms against bacterial and fungal pathogens in the potato?

Considering the key role of cell wall integrity in plant defense and the emerging evidence linking SUT2 to multiple regulatory networks, we hypothesize that *StSUT2* modulates both the biochemical composition and the structural dynamics of the cell wall, thereby influencing disease susceptibility. The disruption of this regulatory axis may impair physical barriers, enhance enzymatic degradation of the cell wall, and render the plant more vulnerable to pathogenic attack.

In the current study, we systematically investigated the effects of *StSUT2* silencing on the cell wall structure and disease resistance in potatoes. We performed an ultrastructural analysis using transmission electron microscopy (TEM) to evaluate cell wall thickness and intercellular space. Additionally, we quantified key cell wall components, including lignin, cellulose, hemicellulose, and pectin, in both leaves and tubers. Enzymatic activities of cell wall-degrading enzymes such as cellulase, polygalacturonase, and xyloglucan endotransglucosylase/hydrolase (XTH) were measured to assess the biochemical implications of *StSUT2* suppression. Finally, we conducted pathogenicity assays using *Fusarium sulphureum* and *Ralstonia solanacearum* to evaluate the disease resistance phenotype of the RNAi lines.

This study provides new insights into the multifaceted role of *StSUT2* in potatoes, linking sucrose transport to structural defense mechanisms and advancing our understanding of how metabolic and structural networks intersect to shape plant–pathogen interactions.

## 2. Results

### 2.1. StSUT2 Silencing Affected Cell Wall Structure

**Figure 1:**
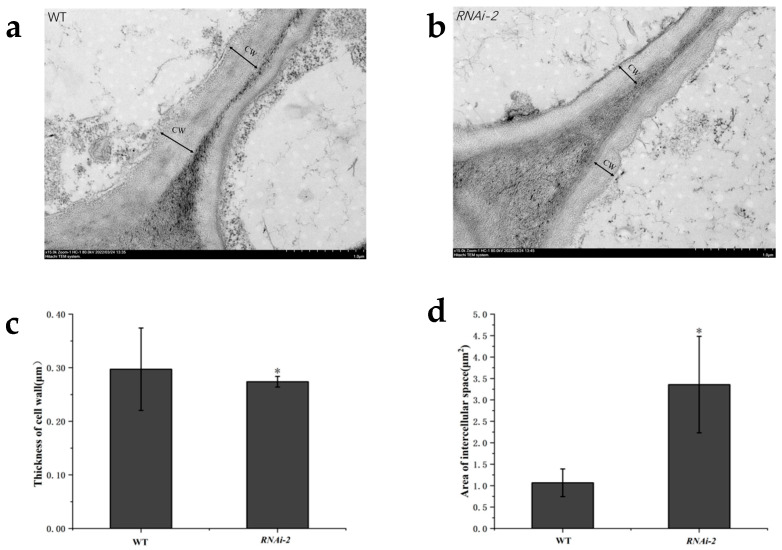
Transmission electron microscopy of the cell wall structure of wild-type and *StSUT2* RNAi lines. (**a**) Cell wall structure of wild-type tuber. (**b**) Cell wall structure of *StSUT2 RNAi-2* tuber. (**c**) Thickness of cell wall. (**d**) Area of the intercellular space. CW: cell wall thickness. *: significant difference compared with WT (*p* < 0.05).

To investigate the potential role of *StSUT2* in regulating potato tuber cell wall architecture, the ultrastructural features of tuber cells were examined in wild type (WT) and the *StSUT2* RNA interference (RNAi) line *RNAi-2* using transmission electron microscopy (TEM) (Figure 1a,b). The results indicated that the cell wall thickness of the *StSUT2 RNAi-2* line was reduced by approximately 7.8% compared to that of the wild type (*p* < 0.05) (Figure 1c). Additionally, the area of intercellular spaces in the *RNAi-2* line was increased by approximately 214% compared to that of the wild type (*p* < 0.05) (Figure 1d). These findings suggest that *StSUT2* may be involved in the regulation of cell wall structure formation.

### 2.2. StSUT2 Silencing Affected the Content of Cell Wall Composition

**Figure 2:**
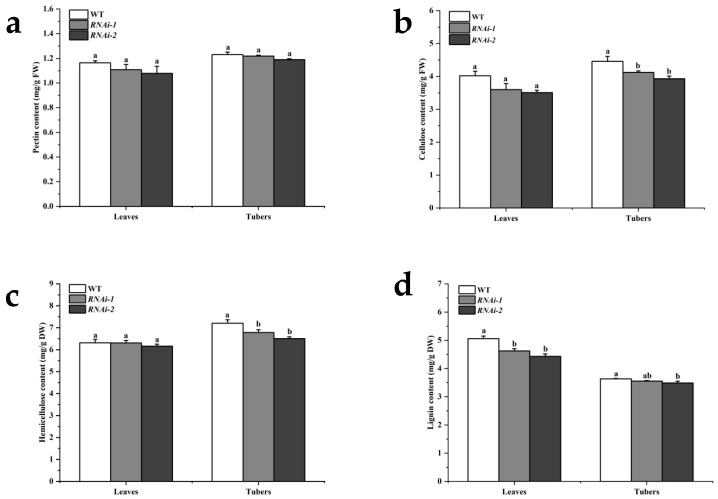
Determination of cell wall component contents in tubers and leaves of wild-type and RNAi lines. (**a**) Content of pectin. (**b**) Content of cellulose. (**c**) Content of hemicellulose. (**d**) Content of lignin. Different letters indicate significant differences among groups, as determined by Duncan’s multiple range test (*p* < 0.05).

Variations in cell wall thickness may be associated with the abundance of cell wall components. Therefore, the contents of pectin, cellulose, hemicellulose, and lignin in the tubers and leaves of *StSUT2* RNAi lines and the wild type (WT) were determined in this study (Table 1). The results showed that, compared with the wild-type line, there was no significant difference in the pectin content in leaves and tubers between the two *StSUT2* RNA interference lines (Figure 2a). The cellulose content decreased by 7.5% and 14.3% in leaves and by 13.8% and 20.1%, respectively, in tubers (Figure 2b). The hemicellulose content showed no significant difference in leaves but decreased by 5.9% and 9.7%, respectively, in tubers (Figure 2c). The lignin content decreased by 8.6% and 12.4% in leaves and by 2.2% and 4.0% in tubers. These findings suggest that the potato *StSUT2* is involved in the regulation of cellulose, hemicellulosem, and lignin accumulation in cell walls, but its effects on source organs (leaves) and sink organs (tubers) are distinct.

### 2.3. StSUT2 Silencing Affected the Activities of Cell Wall Degrading Enzymes

**Figure 3:**
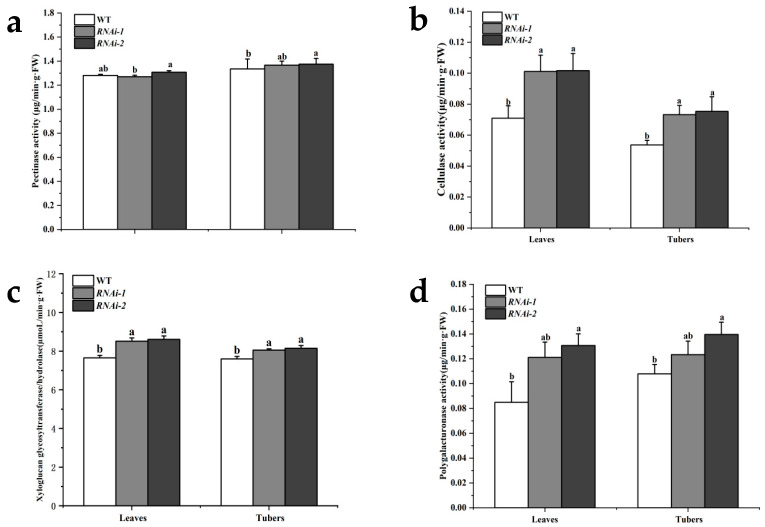
Determination of cell wall-degrading enzyme activities in tubers and leaves of wild-type and RNAi lines. (**a**) Activity of pectinase. (**b**) Activity of cellulase. (**c**) Activity of xyloglucan glycosyltransferase/hydrolase. (**d**) Activity of polygalacturonase. Different letters indicate significant differences among groups, as determined by Duncan’s multiple range test (*p* < 0.05).

The content of plant cell wall components is correlated with the activity of cell wall-degrading enzymes [21]. Therefore, the activities of several cell wall-degrading enzymes, such as pectinase, cellulase, xyloglucan glycosyltransferase/hydrolase (XTH), and polygalacturonase, were determined in the tubers and leaves of two *StSUT2* RNAi lines and the WT (Table 2). The results showed that, compared with the wild type, there was no significant difference in the pectinase activity between the two *StSUT2* RNAi lines (Figure 3a). Cellulase activity increased by 42.4% and 43.3% in leaves and by 36.5% and 40.4%, respectively, in tubers (Figure 3b). XTH activity increased by 11.3% and 12.5% in leaves and by 6.0% and 7.2%, respectively, in tubers (Figure 3c). Polygalacturonase activity increased by 42.6% and 53.9% in leaves and by 14.4% and 29.4%, respectively, in tubers (Figure 3d). These findings suggest that *StSUT2* is involved in regulating the activities of cell wall-degrading enzymes such as cellulase, XTH, and polygalacturonase.

### 2.4. Silencing of StSUT2 Increases Susceptibility to Dry Rot and Bacterial Wilt Pathogens in Potato

The plant cell wall serves as a critical physical and biochemical barrier against pathogen invasion [13,22]. Numerous studies have demonstrated that the integrity and composition of the cell wall are central to plant defense mechanisms, and that modifications to cell wall structure can significantly influence susceptibility to disease [10,11]. To further assess the role of *StSUT2* in potato defense, two major pathogens of potatoes were used to inoculate the leaves and tubers of *StSUT2* RNAi lines and wild-type (WT) plants.

**Figure 4:**
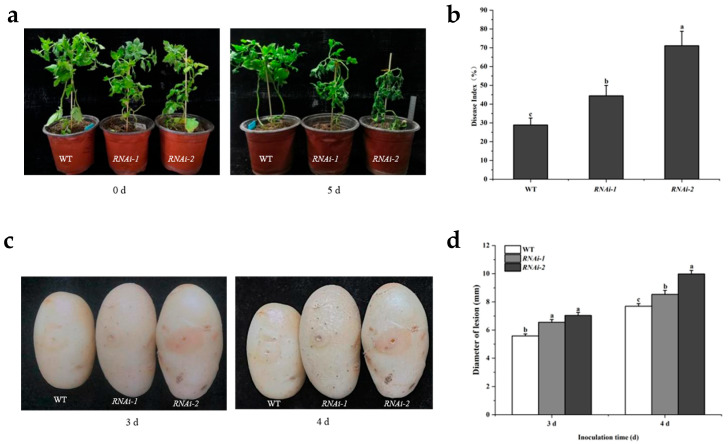
The difference in lesions between wild-type and RNAi lines inoculated with potato bacterial wilt pathogen *Ralstonia solanacearum*. (**a**) The difference between wild-type and RNAi series potted seedlings inoculated with potato bacterial wilt at 0 and 5 days. (**b**) Lesion index of potted plants inoculated with wild type and RNAi strains of potato bacterial wilt. (**c**) Comparison between wild-type and RNAi tuber inoculated with potato bacterial wilt pathogen for 3 days and 4 days. (**d**) Comparison of spot diameter between wild-type and RNAi tuber inoculated with potato bacterial wilt for 3 days and 4 days. Different letters indicate significant differences among groups, as determined by Duncan’s multiple range test (*p* < 0.05).

When potted, seedlings were challenged with the bacterial wilt pathogen *Ralstonia solanacearum*; WT plants exhibited only minor leaf wilting. In contrast, *RNAi-1* lines displayed pronounced leaf crumpling and wilting, while the *RNAi-2* line experienced a complete collapse and severe foliar desiccation (Figure 4a,b). Tuber inoculation with *R. solanacearum* reinforced these observations, as both RNAi lines developed significantly larger necrotic plaques than WT tubers (Figure 4c,d).

**Figure 5:**
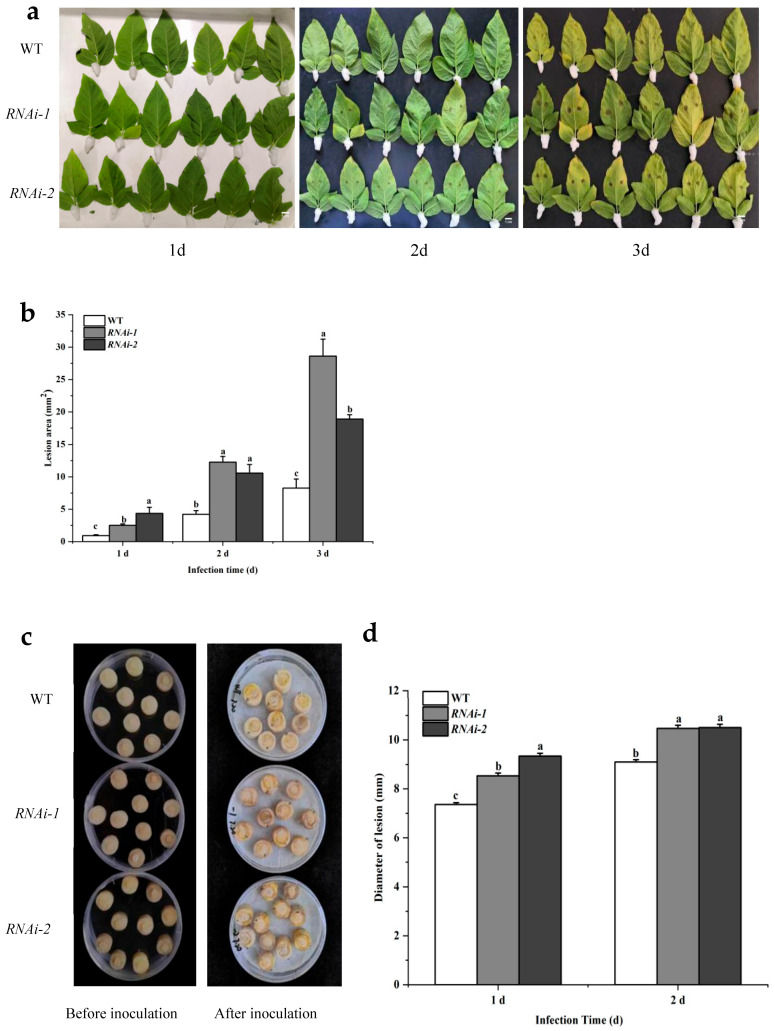
The difference in lesions between wild-type and RNAi lines inoculated with potato dry rot pathogen *Fusarium sulphureum.* (**a**) Differences in disease spots between wild-type and RNAi isolated leaves after inoculation with dry rot bacteria for 1 day, 2 days, and 3 days. (**b**) Comparison of lesion area between wild-type and RNAi strain after 1 day, 2 days, and 3 days of inoculation with dry rot bacteria in isolated leaves. (**c**) Comparison before and after inoculation with the potato dry rot pathogen between the wild-type and the RNAi series. (**d**) Comparison of spot diameter between wild-type and RNAi tubers inoculated with dry rot bacteria 1 and 2 days later. Different letters indicate significant differences among groups, as determined by Duncan’s multiple range test (*p* < 0.05).

Inoculation with the dry rot fungus *Fusarium sulphureum* resulted in markedly larger lesion areas on detached leaves and significantly greater plaque diameters on tubers of both RNAi lines compared to WT plants (Figure 5).

These results, observed consistently across leaf, tuber, and whole-plant infection models, demonstrate that the suppression of *StSUT2* compromises the resistance to both *Fusarium sulphureum* and *Ralstonia solanacearum*. Together, the data highlight *StSUT2*′s essential role in mediating structural defense and limiting pathogen proliferation in the potato.

## 3. Discussion

This study fills a critical research gap by elucidating the role of *StSUT2* in modulating potato cell wall dynamics and immunity, an area previously underexplored compared to its known functions in sucrose transport and developmental processes. By demonstrating that *StSUT2* silencing alters the cell wall composition and increases susceptibility to *Fusarium sulphureum* and *Ralstonia solanacearum*, our findings establish a novel connection between sucrose transport, cell wall integrity, and pathogen defense in the potato. These insights have significant implications for improving crop resilience, as targeting *SUT2* could enhance cell wall-based resistance mechanisms in the potato and related crops, potentially mitigating yield losses due to biotic stresses.

### 3.1. StSUT2 Silencing Induces Changes in Cell Wall Structure and Composition

*SUT2*, initially identified as a sucrose sensor [20], is a low-affinity sucrose transporter [2,21]. Its roles extend beyond carbohydrate transport, with evidence linking *SUT2* to stress signaling and cell wall metabolism [15]. In this study, suppressing the *StSUT2* expression in potatoes caused significant structural and biochemical changes in the cell wall.

Transmission electron microscopy showed reduced cell wall thickness in the *StSUT2* RNAi-2 line compared to the wild type, along with larger intercellular spaces. These changes suggest weaker mechanical support and reduced cellular cohesion. The thinner cell walls align with prior transcriptomic data, indicating downregulated genes for wall biosynthesis in *StSUT2*-silenced lines [15].

Biochemical analysis confirmed these structural component changes, as the lignin, cellulose, and hemicellulose contents were significantly lower in the tubers of RNAi lines, with lignin also reduced in the leaves. These are critical polymers that provide rigidity and resistance to enzymatic degradation [23]. Interestingly, the pectin content remained unchanged, suggesting a selective impact of *StSUT2* silencing on the wall polysaccharide metabolism. In parallel, enzymatic assays demonstrated that the activities of cellulase, polygalacturonase, and xyloglucan endotransglucosylase/hydrolase (XTH) were significantly elevated in both the leaves and tubers of RNAi lines. These enzymes are involved in cell wall loosening and remodeling and are often upregulated during developmental transitions or stress responses. The enhanced enzymatic activity likely contributes to the reduced levels of structural polymers, suggesting an imbalance between synthesis and degradation processes in the context of *StSUT2* silencing.

Comparatively, a study on tomatoes reported that the suppression of *SlSUT2* altered the plant’s symbiotic and defense capabilities via brassinosteroid (BR) signaling modulation [4]. In line with this, BR signaling is known to suppress cell wall-degrading enzymes and promote wall reinforcement [24,25]. We hypothesize that the potato *StSUT2* may similarly interact with BR-regulated pathways, influencing the expression and activity of genes involved in wall metabolism. This would explain the coupled decrease in structural polysaccharides and increase in degrading enzymes. Further protein–protein interaction studies are required to validate this mechanistic link.

### 3.2. StSUT2 Silencing Impairs Resistance to Bacterial and Fungal Pathogens

The structural integrity of the plant cell wall is essential for basal immunity, acting as a first line of defense against pathogen invasion [10]. Alterations in the cell wall composition can either enhance or compromise resistance, depending on the balance between defense activation and wall plasticity [13,21]. In our study, *StSUT2*-silenced lines showed an increased susceptibility to *Fusarium sulphureum* and *Ralstonia solanacearum*, demonstrating the involvement of *StSUT2* in disease resistance mechanisms.

Disease assays consistently showed that RNAi lines exhibited significantly larger lesion areas and higher disease severity scores in leaves and tubers compared to the WT. The more severe collapse observed in *RNAi-2* plants under *R. solanacearum* infection suggests that the extent of *StSUT2* suppression correlates with resistance loss.

These findings are supported by earlier reports where cell wall lignification was directly associated with pathogen resistance. For example, transgenic tobacco plants with reduced lignin content exhibited a higher susceptibility to *Pseudomonas solanacearum* [12], while overexpression of *MdMYB54* in apple enhanced the resistance to *Fusarium solani* by increasing cellulose deposition and regulating the pectate lyase activity [26]. Likewise, the *OsWRKY53*-mediated thickening of sclerenchyma cell walls improved resistance to *Xanthomonas oryzae* in rice [13].

Given the reduced lignin, cellulose, and hemicellulose levels in the *StSUT2* RNAi lines, it is plausible that weakened physical barriers allowed for easier pathogen ingress and colonization. Simultaneously, elevated cell wall-degrading enzyme activity may have facilitated the cell wall breakdown during infection, further enhancing susceptibility.

The distinct responses observed between leaves (source organs) and tubers (sink organs) in *StSUT2*-silenced lines likely reflect their differing roles in sucrose allocation and cell wall metabolism. Leaves, as primary sites of photosynthesis, rely on *StSUT2* to regulate sucrose transport, which influences the availability of carbon precursors for cell wall components such as cellulose and lignin. In contrast, tubers, as storage organs, prioritize carbohydrate storage over structural reinforcement, potentially leading to a greater reliance on hemicellulose for cell wall integrity. The pronounced susceptibility in RNAi tubers, with larger necrotic plaques and lesion diameters, suggests that *StSUT2* silencing disrupts the hemicellulose deposition more severely in sink organs, compromising their ability to limit pathogen spread. This is consistent with studies showing that sink organs often exhibit an altered cell wall composition under stress, as seen in Arabidopsis roots with reduced cellulose synthase activity, which increased the susceptibility to fungal pathogens [27]. Furthermore, the differential expression of cell wall-modifying enzymes, such as expansins and pectinases, between source and sink tissues may exacerbate susceptibility in tubers by facilitating pathogen-induced wall degradation [27].

In this study, pathogen resistance was primarily assessed through lesion size and disease index measurements, which provided reproducible and consistent evidence of enhanced susceptibility in RNAi plants. However, integrating quantitative assays such as a qPCR-based pathogen load estimation for *R. solanacearum* and fungal biomass quantification for *F. sulphureum* in future studies would provide a more direct measure of pathogen colonization and spread. Such approaches would not only validate the phenotypic observations but also strengthen the mechanistic understanding of how *StSUT2* influences pathogen resistance. Additionally, to explore the potential link between *StSUT2* and brassinosteroid signaling, future experiments could involve applying brassinosteroid inhibitors or exogenous brassinosteroids to *StSUT2* RNAi lines to assess their impact on cell wall composition and pathogen resistance.

### 3.3. Comparative Roles of SUT2 in Potatoes and Other Crops: Implications for Crop Improvement

The role of *StSUT2* in potato cell wall metabolism and pathogen resistance shares both similarities and differences with *SUT2* homologs in other agriculturally important crops, placing our findings in a broader plant physiology and crop-improvement context. In a closely related solanaceous crop (tomato), *SUT2* modulates brassinosteroid (BR) signaling, influencing the cell wall composition and defense responses against fungal pathogens [4]. Similarly to our observations in the potato, the silencing of this *SUT2* homolog led to reduced lignin and cellulose levels, compromising the resistance to necrotrophic pathogens. However, unlike potatoes, where *StSUT2* silencing significantly impacted hemicellulose content in tubers, minimal changes in hemicellulose were reported in this crop, suggesting species-specific differences in the *SUT2*-mediated regulation of cell wall polysaccharides. In a monocot crop, *SUT2* is primarily localized in sink tissues and regulates sucrose partitioning to support grain filling, but its role in biotic stress is less pronounced [28]. Mutants of this *SUT2* homolog exhibit an altered cell wall composition in the leaves, with reduced cellulose and increased xyloglucan, correlating with the enhanced susceptibility to bacterial pathogens [29]. In another major cereal crop, *SUT2* plays a critical role in sucrose transport to sink tissues, such as developing kernels, and its silencing results in reduced cell wall thickness and lignin content, leading to the increased susceptibility to fungal pathogens [7]. This mirrors the potato phenotype, where weakened cell walls in *StSUT2* RNAi lines facilitated pathogen ingress. In a different cereal crop, *SUT2* influences the sucrose allocation to developing grains and has been linked to cell wall reinforcement in response to biotic stress, with mutants showing reduced cellulose and hemicellulose levels and a heightened susceptibility to fungal pathogens [30]. Unlike the potato, where *StSUT2* impacts both the source and sink organs, this *SUT2* primarily affects sink tissues, indicating tissue-specific roles. These comparative findings suggest a conserved function for *SUT2* proteins in modulating the cell wall integrity and pathogen resistance across monocots and dicots, though the extent of their impact varies by species and tissue type. For crop improvement, manipulating the *SUT2* expression offers promising avenues. Overexpressing *StSUT2* in the potato or its homologs in other crops could strengthen cell walls to enhance resistance to necrotrophic pathogens, while in cereals, the targeted modulation of *SUT2* could balance yield and biotic stress tolerance. These insights highlight *SUT2* as a versatile target for engineering pathogen-resistant crops, with strategies designed to species-specific physiological roles and stress responses [9].

Together, these results support the hypothesis that *StSUT2* contributes to potato immunity by maintaining cell wall structural integrity and restricting excessive degradation. The dual impact on both wall biosynthesis and enzyme regulation positions *StSUT2* as a key integrator of metabolic and defense pathways. Future studies using overexpression systems and BR pathway mutants could provide deeper insights into how sucrose transporters intersect with hormonal and defense signaling in potatoes.

## 4. Materials and Methods

### 4.1. Plant Materials and Pathogenic Bacteria

#### 4.1.1. Plant Materials

The potato tetraploid cultivation variety ‘Shepoty’ wild type (WT) and its *StSUT2* downregulated expression lines *RNAi-1* (43.4% downregulated expression) and *RNAi-2* (62.9% downregulated expression) were used in this study. All the above materials are preserved in our laboratory. The selection of the ‘Shepoty’ variety is reasonable, as it has high sucrose transport efficiency, a tetraploid genetic background consistent with the main potato varieties in production, and moderate sensitivity to target pathogens. All materials were propagated on 3% MS medium then cultured in a growth chamber (16 h light/8 h dark, 25 ± 2 °C, and 30% relative air humidity) for 30 days. After that, they were transplanted into plastic pots in a greenhouse, where the substrate was a 1:1 mixture of nutrient soil and vermiculite. The materials continued to grow for 90 days under the same light and temperature conditions as in the growth chamber, and finally, minitubers were harvested.

#### 4.1.2. Pathogen Materials

*Fusarium sulphureum*, a dry rot pathogen, was generously provided by Professor Yang Bi of Gansu Agricultural University, China; *Ralstonia solanacearum*, a bacterial wilt pathogen (XG5), was donated by Professor Huilan Chen from Huazhong Agricultural University, China. The pathogenicity of the above two pathogenic materials has been verified before inoculation.

### 4.2. Determination of Cell Wall Structure

The minitubers were cut into 1 mm × 1 mm thin slices and fixed with electron microscope fixative. Observations and image capture were performed using a transmission electron microscope. Cell wall thickness and intercellular space area were measured using Image-Pro Plus 6.0 software, calibrated with the image scale.

### 4.3. Pectin Content Determination

Pectin content was quantified using an improved carbazole sulfuric acid spectrophotometric method [31]. Approximately 1.0 g of tissue, previously frozen in liquid nitrogen, was ground into a fine powder. The powder was transferred to a 20 mL centrifuge tube, submerged in 75% ice-cold ethanol, and mixed thoroughly. The mixture was incubated in an ice bath for 20 min, centrifuged at 10,000 rpm for 10 min, and the supernatant was discarded. The pellet was washed with ice-cold acetone, followed by centrifugation under the same conditions. The pellet was then extracted with a 1:1 methanol/chloroform solution, followed by a final methanol precipitation, and the resulting cell wall material was freeze-dried. A 2.0 mg portion of the dried cell wall material was weighed into a test tube, combined with 15 μL of ammonium oxalate buffer, and incubated in boiling water for 1 h. After cooling, the sample was centrifuged at 12,000 rpm for 5 min, and the supernatant was collected. This extraction was repeated three times, with supernatants combined. To 20 μL of the combined supernatant, 1.2 mL of 0.0125 M H_2_SO_4_-borax solution was added, mixed thoroughly, and heated in boiling water for 5 min. After cooling to room temperature, 20 μL of 0.15% carbazole was added, and the mixture was allowed to stand for 20 min. Absorbance was measured at 520 nm, and pectin content was calculated using a galacturonic acid standard curve, expressed as μg/g fresh weight (FW). Each sample was analyzed in triplicate.

### 4.4. Analysis of Cellulose Content

Cellulose content was quantified using an improved version of the method described by Zhao Yijie [32]. A 1.0 g sample, previously frozen in liquid nitrogen, was ground into a fine powder and transferred to a centrifuge tube. An acetic acid/nitric acid reagent (80% acetic acid) was added, and the mixture was thoroughly mixed and cooled. The samples were centrifuged at 10,000 rpm for 30 min, and the supernatant was discarded. The pellet was washed with distilled water, followed by the addition of 6 mL of 67% sulfuric acid, and the mixture was agitated for 1 h. A 0.1 mL aliquot of the resulting supernatant was combined with 4 mL of distilled water and 10 mL of pre-chilled anthrone–sulfuric acid reagent (0.2 g anthrone in 100 mL sulfuric acid). The mixture was heated in boiling water for 10 min and cooled. Absorbance was measured at 620 nm, and cellulose content was calculated using a standard curve, expressed as mg/g. Each sample was analyzed in triplicate.

### 4.5. Hemicellulose Content Determination

Hemicellulose content was determined following the method described by Liu Xingju and Sun Qing [33,34]. A 20 mg portion of dried sample was treated with 1 mL of 80% calcium nitrate to remove starch. The mixture was incubated at 90 °C for 10 min and centrifuged at 8000 rpm for 10 min at 25 °C, with the supernatant discarded. The pellet was washed with distilled water three times, followed by drying at 105 °C. The dried pellet was hydrolyzed with 200 μL of 2 M HCl in a 90 °C water bath for 1 h. After cooling, 200 μL of 1 M NaOH was added, and the mixture was centrifuged at 8000 rpm for 10 min. A 200 μL aliquot of the supernatant was combined with 150 μL of 1% 3,5-dinitrosalicylic acid and 650 μL of distilled water, incubated at 90 °C for 5 min, cooled, and centrifuged again. Absorbance was measured at 540 nm. Hemicellulose content was calculated using the following formula: hemicellulose (mg/g dry weight) = 2.02 × (ΔA + 0.0043)/W, where ΔA is the absorbance and W is the sample weight. Each sample was analyzed in triplicate.

### 4.6. Determination of Lignin Content

Lignin content was quantified using the acetyl bromide method described by Sun Qing and Fukushima et al. [33]. Samples were dried at 45 °C to a constant weight and ground into a fine powder. Approximately 15 mg of the powdered sample was treated with 25% acetyl bromide and perchloric acid. The mixture was incubated in a 70 °C water bath for 40 min, with agitation every 10 min. After cooling to room temperature, sodium hydroxide and hydroxylamine hydrochloride were added, and the mixture was thoroughly mixed. A 10 μL aliquot of the supernatant was diluted to 2 mL with glacial acetic acid and absorbance was measured at 280 nm. Lignin content was calculated using the following formula: lignin (mg/g dry weight) = 0.0735 × (ΔA − 0.0068)/(W × T), where ΔA is the absorbance, W is the sample mass in grams, and T is the dilution factor. Each sample was analyzed in triplicate.

### 4.7. Determination of Pectinase (PME) Activity

Pectinase (PME) activity was quantified following the method described by Ma Jing et al. [35]. A 1.0 g sample of fresh tissue was frozen in liquid nitrogen, ground into a fine powder, and extracted in 5.0 mL of 1 M cold NaCl solution containing 1% polyvinylpolypyrrolidone (PVPP) at 4 °C for 4 h. The extract was centrifuged at 10,000 rpm for 30 min at 4 °C, and the supernatant’s pH was adjusted to 7.5 using 0.01 M NaOH. A reaction mixture was prepared, consisting of 100 μL of enzyme extract, 600 μL of 0.5% pectin (pH 7.5), 150 μL of 0.01% bromothymol blue, and 100 μL of distilled water. Absorbance was measured at 620 nm over 10 min. PME activity was calculated using a hydrochloric acid standard curve and expressed as μg/min·g fresh weight (FW). Each sample was analyzed in triplicate.

### 4.8. Cellulase (CEL) Activity Assay

Cellulase (CEL) activity was quantified using a modified method based on Yuan Li [36]. A 1.0 g sample of fresh tissue was frozen in liquid nitrogen, ground into a fine powder in a pre-cooled mortar, and transferred to a 10 mL centrifuge tube. The powder was suspended in 5.0 mL of pre-cooled 50 mM acetic acid buffer (pH 4.5) containing 7.5% NaCl and 2.5% polyvinylpyrrolidone (PVP). The suspension was agitated on a shaker and centrifuged at 10,000 rpm for 30 min at 4 °C. The supernatant, serving as the crude enzyme solution, was collected. CEL activity was measured following methods described by Carvajal and Zhang et al. [37,38]. A 0.1 mL aliquot of the crude enzyme extract was combined with 1.5 mL of 10 g/L carboxymethylcellulose (CMC) and incubated at 37 °C for 1 h. The reaction was terminated by adding 1.5 mL of 3,5-dinitrosalicylic acid (DNS) reagent, followed by boiling for 5 min, cooling, and dilution to 25 mL with distilled water. A blank control was prepared using acetic acid buffer (pH 4.5) and DNS reagent. Absorbance was measured at 540 nm, and CEL activity was calculated using a glucose standard curve, expressed as μg/min·g fresh weight (FW). Each sample was analyzed in triplicate.

### 4.9. Determination of Xyloglucan Glycosyltransferase/Hydrolase (XTH) Activity in Plant Xyglucan

The activity of XTH was determined by using the Plant XTH enzyme-linked immunosorbent Assay Kit (Catalog number: XTH-ELISA-001, PlantBioTech, Lanzhou, China), expressed as μg/min·g fresh weight (FW).

### 4.10. Determination of Polygalacturonidase (PG) Activity

Polygalacturonidase (PG) activity was quantified following the method described by Yuan Li et al. [39]. The crude enzyme solution was extracted using the same procedure as for cellulase (CEL) activity, as outlined in Section 4.8. A 100 μL aliquot of the crude enzyme extract was preheated at 37 °C for 3 min. Subsequently, 500 μL of preheated 1.0% polygalacturonic acid (pH 4.0) and 1.40 mL of acetic acid buffer (pH 4.0) were added to the extract. The mixture was incubated at 37 °C for 1 h, after which 1.5 mL of 3,5-dinitrosalicylic acid (DNS) reagent was added to terminate the reaction. The solution was boiled for 5 min, cooled to room temperature, and diluted to 25 mL with distilled water. A control was prepared by boiling a mixture of 1.50 mL acetic acid buffer (pH 4.5), 0.50 mL polygalacturonic acid, and 1.5 mL DNS reagent for 5 min. Absorbance was measured at 540 nm. PG activity was calculated using a D-(+)-galacturonic acid standard curve, with one unit (U) of pectinase activity defined as the amount of enzyme producing 1 μg of free galacturonic acid per gram of fresh sample per minute. Activity was expressed as μg/min·g fresh weight (FW). Each sample was analyzed in triplicate.

### 4.11. Identification of Pathogen Resistance

The resistance of potted seedlings was assessed using Cellier’s root injury grafting method [40]. Minor cuts were made to the roots of 4-week-old seedlings. A volume of 10 mL of *Ralstonia solanacearum* suspension with a concentration of OD_600_nm = 0.1 was applied at the wound site, and an equal volume of sterilized water was used as a control. The seedlings were incubated at 28 °C. After approximately one week, wilting symptoms were observed, graded, and the disease index was calculated using the same method as for tube-seedling inoculation. Wilting severity was classified into five levels: Level 0, no wilting; Level 1, 1–25% leaf wilting; Level 2, 26–50%; Level 3, 51–75%; and Level 4, more than 75% leaf wilting. The disease index (DI) was calculated using the following formula: DI = 100 × (n_1_ × 1 + n_2_ × 2 + n_3_ × 3 + n_4_ × 4)/[(n_0_ + n_1_ + n_2_ + n_3_ + n_4_) × 4]. For each material, 3 pots were inoculated each time as technical replicates, and inoculations were performed three times or more as biological replicates.

Whole-potato resistance was assessed following Zhang Wunwei’s perforated inoculation method [41]. Tubers were sterilized, UV-irradiated for 10 min, and kept in sterile conditions. A bacterial suspension was prepared from Petri dish cultures and adjusted to the appropriate concentration using a hemocytometer. Tubers were punctured at the equator with a sterile needle, and 10 μL of the *Ralstonia solanacearum* suspension was inoculated into each hole. Tubers were sealed in plastic bags and incubated at 25 ± 2 °C. Lesion diameters were measured daily using a vernier caliper. Three potato tubers were used per treatment, with three replicates performed.

Leaf resistance was evaluated based on Zhang Weina’s in vitro leaf inoculation method [42]. Leaves of uniform growth stage (5–8 weeks old) were selected. A 10 μL droplet of *Fusarium sulphureum* suspension was spotted on the abaxial side of each leaf. Leaves were placed in inoculant trays and incubated at 22 °C under a 16 h light/8 h dark photoperiod. Disease progression was monitored daily, and lesion areas were quantified using ImageJ 1.54. Software.

Tuber resistance was measured using the bacterial cake inoculation method described by Yang Zhimin [43]. Potato tubers were cored using a sterile punch and cut into round slices (1 cm thick, 1 cm diameter). The center of each slice was inoculated with a 6.5 mm *Fusarium sulphureum* plug cultured for one week under conditions of 23–25 °C and 72–75% relative humidity. Lesion diameters were measured daily. Each treatment included 10 slices, with three replicates performed.

### 4.12. Data Processing

All experimental data in this paper were statistically analyzed using Excel 2023, graphed using Origin 2024 software, one-way ANOVA was performed using IBM SPSS Statistics 28, and differences were analyzed using Duncan multiple comparisons (*p* < 0.05).

## 5. Conclusions

In summary, the silencing of StSUT2 leads to reduced cell wall thickness, increased intercellular spaces, decreased contents of lignin, cellulose, and hemicellulose, and elevated activity of cell wall-degrading enzymes, which may consequently weaken the plant’s resistance to fungal and bacterial pathogens. While our findings clearly highlight the structural and biochemical consequences of StSUT2 silencing, the precise molecular mechanism underlying these changes remains to be elucidated. Future studies incorporating transcriptomic profiling and the analyses of hormone signaling pathways, particularly potential interactions with brassinosteroids (BRs), will be essential to validate and strengthen the mechanistic link between the StSUT2 function and cell wall remodeling. In addition, field trials with StSUT2-overexpressing lines could further validate the potential for manipulating StSUT2 to develop pathogen-resistant potato varieties, contributing to sustainable agriculture.

The role of StSUT2 in regulating cell wall integrity and pathogen resistance in the potato has significant implications for crop improvement, particularly in addressing biotic stresses that threaten global potato production. By demonstrating that StSUT2 modulates cell wall composition and influences the susceptibility to pathogens like *Fusarium sulphureum* and *Ralstonia solanacearum*, this study shows its potential as a target for breeding programs aimed at developing disease-resistant potato varieties. Enhancing StSUT2 expression could strengthen cell wall-based defense mechanisms, reduce yield losses, and improve tuber quality in regions affected by bacterial wilt and dry rot. These findings also suggest that SUT2 homologs in other crops could be similarly leveraged, offering a versatile strategy for improving resilience across diverse agricultural systems. Future breeding efforts could integrate StSUT2 manipulation with genomic selection to accelerate the development of robust potato cultivars, contributing to sustainable agriculture and food security.

## Figures and Tables

**Table 1:** The contents of cell wall components in tubers and leaves of wild-type and RNA interference (RNAi) lines.

Line	Organ	Pectin Content (mg/g FW)	Cellulose Content (mg/g FW)	Hemicellulose Content (mg/g DW)	Lignin Content (mg/g DW)
WT	Leaves	1.16	4.02	6.31	5.06
	Tubers	1.23	4.46	7.21	3.63
*RNAi-1*	Leaves	1.11	3.60	6.31	4.63
	Tubers	1.22	4.12	6.78	3.56
*RNAi-2*	Leaves	1.08	3.51	6.16	4.43
	Tubers	1.19	3.93	6.51	3.49

**Table 2:** The activities of cell wall-degrading enzymes in tubers and leaves of wild-type and RNA interference (RNAi) lines.

Line	Organ	Pectinase Activity (μg/min·g·FW)	Cellulase Activity (μg/min·g·FW)	Xyloglucan Glycosyltransfer-ase/Hydrolase Activity (µmoL/min·g·FW)	Polygalacturonase Activity (μg/min·g·FW)
WT	Leaves	1.28	0.07	7.65	0.08
	Tubers	1.34	0.05	7.60	0.11
*RNAi-1*	Leaves	1.27	0.10	8.52	0.12
	Tubers	1.37	0.07	8.05	0.12
*RNAi-* *2*	Leaves	1.31	0.10	8.61	0.13
	Tubers	1.37	0.08	8.14	0.14

## Data Availability

The original contributions presented in this study are included in the article.
